# Efficacy and Safety of Mesenchymal Stem Cell Transplantation in the Treatment of Autoimmune Diseases (Rheumatoid Arthritis, Systemic Lupus Erythematosus, Inflammatory Bowel Disease, Multiple Sclerosis, and Ankylosing Spondylitis): A Systematic Review and Meta-Analysis of Randomized Controlled Trial

**DOI:** 10.1155/2022/9463314

**Published:** 2022-03-24

**Authors:** Liuting Zeng, Ganpeng Yu, Kailin Yang, Wang Xiang, Jun Li, Hua Chen

**Affiliations:** ^1^Department of Rheumatology and Clinical Immunology, Peking Union Medical College Hospital, Chinese Academy of Medical Sciences & Peking Union Medical College, Beijing, China; ^2^People's Hospital of Ningxiang City, Ningxiang city, Hunan Province, China; ^3^Beijing Anzhen Hospital, Capital Medical University, Beijing, China; ^4^Zhangjiajie City People's Hospital, Zhangjiajie, Hunan, China

## Abstract

**Objective:**

To evaluate the efficacy and safety of mesenchymal stem cell (MSC) transplantation in the treatment of autoimmune diseases.

**Methods:**

The Chinese and English databases were searched for clinical research on the treatment of autoimmune diseases with mesenchymal stem cells. The search time range is from a self-built database to October 1, 2021. Two reviewers independently screened the literature according to the inclusion and exclusion criteria, extracted data, and evaluated the bias of the included studies. RevMan 5.3 analysis software was used for meta-analysis.

**Results:**

A total of 18 RCTs involving 5 autoimmune diseases were included. The 5 autoimmune disease were rheumatoid arthritis (RA), systemic lupus erythematosus (SLE), inflammatory bowel disease, ankylosing spondylitis, and multiple sclerosis. For RA, the current randomized controlled trials (RCTs) still believe that stem cell transplantation may reduce disease activity, improve the clinical symptoms (such as DAS28), and the percentage of CD4+CD 25+Foxp3+Tregs in the response group increased and the percentage of CD4+IL-17A+Th17 cells decreased. The total clinical effective rate of RA is 54%. For SLE, the results showed that mesenchymal stem cell transplantation may improve SLEDAI [-2.18 (-3.62, -0.75), *P* = 0.003], urine protein [-0.93 (-1.04, -0.81), *P* < 0.00001], and complement C3 [0.31 (0.19, 0.42), *P* < 0.00001]. For inflammatory bowel disease, the results showed that mesenchymal stem cell transplantation may improve clinical efficacy [2.50 (1.07, 5.84), *P* = 0.03]. For ankylosing spondylitis, MSC treatment for 6 months may increase the total effective rate; reduce erythrocyte sedimentation rate, intercellular adhesion molecules, and serum TNF-*α*; and improve pain and activity. For multiple sclerosis, the current research results are still controversial, so more RCTs are needed to amend or confirm the conclusions. No obvious adverse events of mesenchymal stem cell transplantation were found in all RCTs.

**Conclusion:**

MSCs have a certain effect on different autoimmune diseases, but more RCTs are needed to further modify or confirm the conclusion.

## 1. Introduction

Autoimmune diseases are a series of diseases caused by the immune system's response to self-antigens, resulting in self-tissue damage or dysfunction [1]. It mainly includes systemic lupus erythematosus (SLE), rheumatoid arthritis (RA), Sjogren's syndrome, polymyositis and dermatomyositis [1, 2]. Many autoimmune diseases are characterized by the production of autoantibodies, which bind to the host's own proteins or form immune complexes and deposit in tissues. Any organ of the body may become a target organ for autoimmunity, including skin, joints, kidneys, and blood vessels. The inflammatory effect caused by autoantibodies is mediated by binding to Fc receptors on leukocytes, which is an important cause of downstream tissue damage [3, 4]. Meanwhile, autoantibodies can also directly mediate tissue damage in diseases through complement activation [5]. In the development stage of the disease, genetic factors and environmental factors may interact in turn to promote the development of autoimmunity and ultimately lead to tissue inflammation and damage, becoming a chronic disease with multiple organs and multiple system damage [6, 7].

Clinically, once diagnosed, patients should be treated with medication in time to avoid further development of the disease causing damage to organs or systems such as the liver and kidney [8]. Clinically, commonly used glucocorticoids and traditional disease-improving antirheumatic drugs (DMARDs) have good anti-inflammatory, pain-relieving, and improving or delaying disease progression effects and are still used as the first-line choice for clinical treatment of rheumatic immune diseases [9, 10]. However, for first-line treatments with single or combined regimens that do not respond well or cannot tolerate them, other treatment options with potential curative effects need to be considered [11–13]. For example, stem cell transplantation, biological preparations, or new botanical preparations, as well as some antirheumatic drug candidates that may modulate or suppress immunity, in order to alleviate the condition of patients with refractory rheumatism and improve their quality of life. Among them, mesenchymal stem cells are one of the most promising therapeutic strategies [14].

Since the discovery of mesenchymal stem cells (MSC), the understanding of them has continued to deepen. Because of its proliferation and differentiation ability, the medical community expects it to be used in the treatment of clinical diseases [14]. In the past few decades, the initial research on MSC focused on its differentiation ability, but with the discovery of its immunomodulatory function, the direction of MSC-based therapeutic research has changed from the initial regenerative medicine to autoimmune diseases [15]. So far, there have been many reports in the literature on the treatment of autoimmune diseases with MSC [16, 17], and there are more and more researches on the application of MSC in clinical trials. One-third of the clinical studies focused on the treatment of autoimmune diseases by MSC [18]. Studies have found that MSCs are weakly immunogenic and did not express major histocompatibility complex (MHC) class II molecules, apoptotic gene ligands, and T cell costimulatory molecules (B7-1, B7-2, CD40, and CD40L). It did not express or express MHC class I molecules at very low levels, did not induce an immune response in vitro, and also had an immunosuppressive effect. These studies have laid the theoretical foundation for the transplantation of allogeneic bone marrow MSCs to treat autoimmune diseases [19]. For some autoimmune diseases (such as rheumatoid arthritis (RA) and SLE, Crohn's disease, primary Sjogren's syndrome, systemic sclerosis, dermatomyositis, ankylosing spondylitis, psoriasis, multiple sclerosis), animal experiments and clinical controlled trials have also shown that MSC transplantation can improve the clinical symptoms of the above diseases [18–20]. Due to the relative uncontrollability of cell culture used in these clinical randomized controlled trials, the complexity of clinical trial design, and the implementation factors of effective evaluation measures, there is an urgent need for a comprehensive systematic review and meta-analysis of the clinical controlled trials of mesenchymal stem cells for the treatment of autoimmune diseases. Therefore, this study registered the protocol on PROSEPRO in order to provide a complete and comprehensive evaluation and provide new evidence for clinical practice.

## 2. Materials and Methods

### 2.1. Protocol

This systematic review and meta-analysis were conducted strictly in accordance with the protocol registered in PROSPERO (CRD42021277144) and PRISMA guidelines (see supplementary materials) [21].

### 2.2. Research Databases

Chinese databases (including CNKI, VIP database, Wanfang Database, and Sinomed) and English databases (including Embase, Medline, PubMed, and Web of Science) were searched from the establishment of the database to Oct. 1st, 2021. Cochrane Library and ClinicalTrials.gov were also searched. The research retrieval strategy of Embase and PubMed were shown in Table S1^1^.

### 2.3. Search Criteria

#### 2.3.1. Participants

Patients who have been diagnosed with any kind of autoimmune disease according to authoritatively recognized standards were included. Patients had no restrictions on gender, age, region, etc.

#### 2.3.2. Intervention Methods

The intervention of the experimental group was mesenchymal stem cell (MSC), which can be used alone or in combination with other therapies. The intervention measures of the control group were non-MSC therapy, which could be traditional therapy, placebo, etc.

#### 2.3.3. Outcomes

Outcomes were the efficacy and safety indicators of the corresponding disease.

#### 2.3.4. Study design

The randomized controlled trial (RCT) without any restrictions was selected.

#### 2.3.5. Exclusion Criteria

Exclusion criteria include (1) animal experiments, (2) basic research, (3) not RCT, and (4) the intervention of the control group was MSC transplantation.

### 2.4. Research Screening, Extraction, and Quality Assessment

First, we deduplicate the literature according to the title, author, year of publication, etc. Then, we read the abstract for further screening and finally read the full text and further screened according to the literature screening criteria and extracted data from the included RCTs [22]. The risk of bias was independently assessed by two researchers in accordance with the Cochrane Risk Bias Assessment Form [23] provided by the Cochrane Collaboration. Two researchers independently screened the literature, extracted data, and assessed the quality of RCTs. If there is a disagreement, they will negotiate with the third researcher.

### 2.5. Statistical Analysis

Revnan5.3 was used for meta-analysis [22]. Relative risk (RR) and mean difference (MD) are used as the combined effect size of dichotomous variables (such as adverse events and effective rate) and continuous variables (such as SLEDAI score), respectively. The heterogeneity between RCTs was tested by chi-square test, and the test standard was *P* < 0.1. The degree of heterogeneity was judged based on *I*^2^. When *I*^2^ > 50%, it indicated that there is high heterogeneity, and a random effect model was established. On the contrary, when *I*^2^ < 50%, a fixed effect model was established.

## 3. Results

### 3.1. Search Results

**Figure 1:**
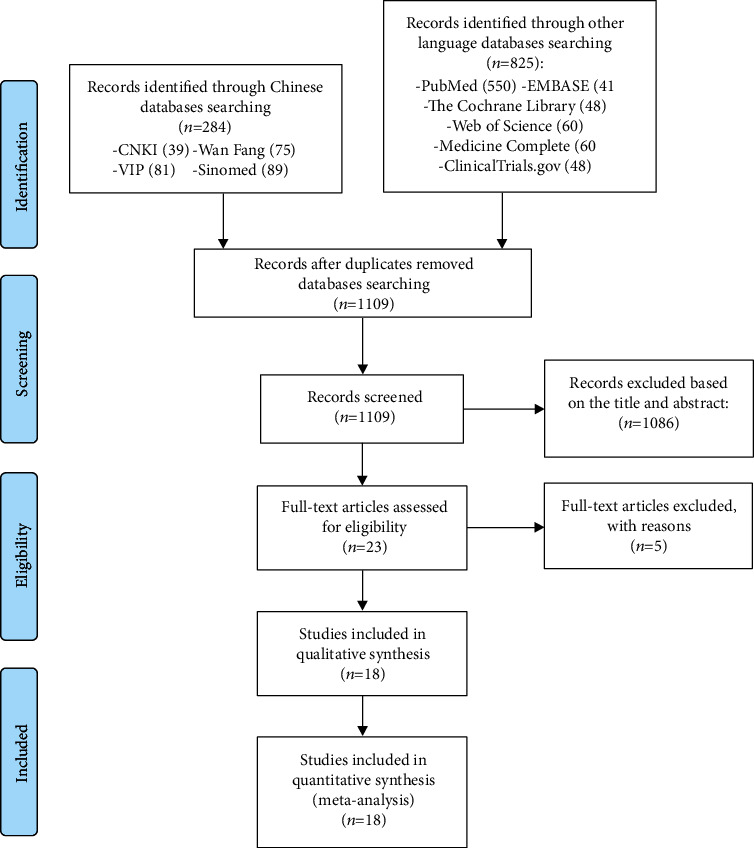
Flow diagram of research screening (CNKI: China National Knowledge Infrastructure).

A total of 1109 records were retrieved initially, and 23 records were left for further screening after preliminary screening. Finally, 18 RCTs were included for they meet the search criteria, while 5 records were excluded [24–28]. The literature screening process is shown in Figure 1.

### 3.2. Description of Included Trials

The included RCTs involved a total of 5 autoimmune diseases: rheumatoid arthritis, systemic lupus erythematosus, inflammatory bowel disease, ankylosing spondylitis, and multiple sclerosis. The studies of Fernández et al. (2018) and Lublin et al. (2014) were divided into 2 subgroups according to the dose, and the study of Petrou et al. (2020) was divided into 2 subgroups according to the route of administration. The included study characteristics were shown in Table 1.

### 3.3. Risk of Bias Assessment

**Figure 2:**
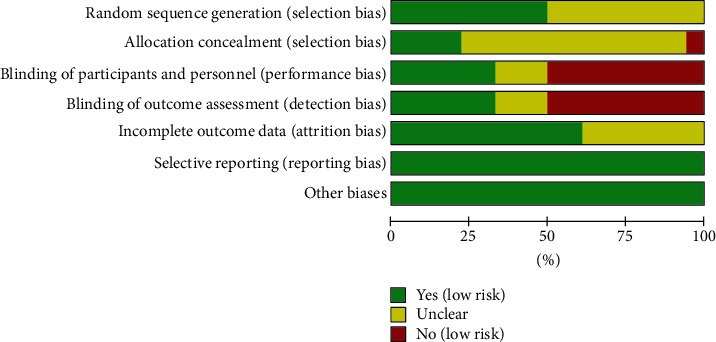
Risk of bias graph.

**Figure 3:**
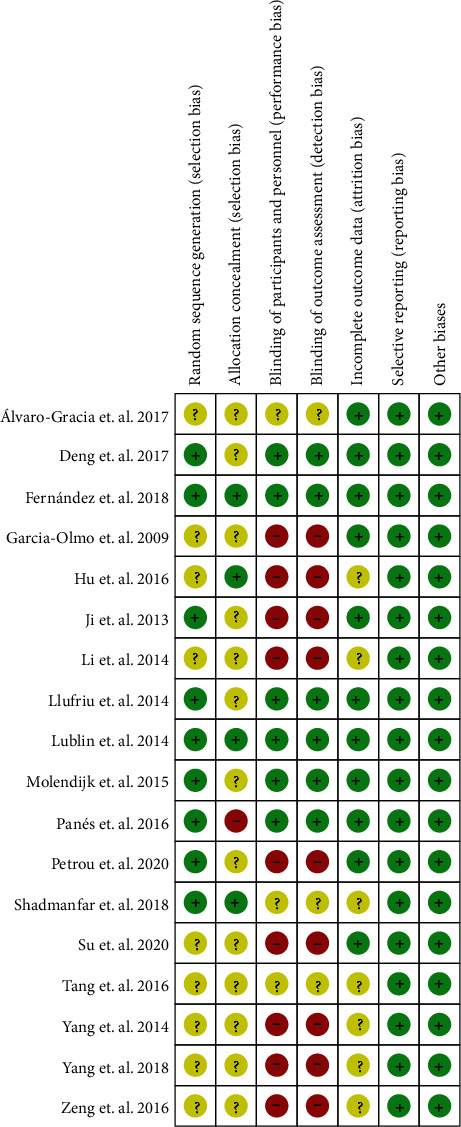
Risk of bias summary.

The summary and graph of risk of bias were shown in Figures 2 and 3.

#### 3.3.1. Random Sequence Generation and Allocation Concealment

Nine RCTs [30, 34, 38, 39, 41–43, 45, 46] described the random sequence generation methods and were rated as low risk of bias. Other RCTs did not describe the random sequence generation method and were assessed as unclear risk of bias. Four RCTs [30, 36, 42, 45] describe allocation concealment methods and were assessed as low risk of bias. Panés et al. [38] did not perform allocation concealment and was assessed as high risk of bias. Other RCTs did not describe the allocation concealment methods and were assessed as unclear risk of bias.

#### 3.3.2. Blinding, Incomplete Outcome Data, and Selective Reporting

Six RCTs [34, 38, 39, 42, 45, 46] describe the specific methods of blind implementation and were assessed as low risk. Álvaro-Gracia et al. [31] and Tang et al. [32] claimed to use the blind method but did not describe the implementation process; Shadmanfar et al. [30] did not mention whether to use blinding; therefore, they were rated as unclear risk of bias. Other RCTs did not use blinding and their outcomes were subjective indicators; hence, they were rated as high risk of bias. Six RCTs [29–31, 33, 36, 44] have missing data and did not use appropriate statistical treatment method; hence, they were rated as unclear risk of bias.

### 3.4. Other Potential Bias

Other sources of bias were not observed, and they were rated as low risk of bias.

### 3.5. Rheumatoid Arthritis

RA often manifests as joint swelling, joint stiffness, and tenderness in the morning. It is mainly due to the invasion and damage of the cartilage and bone due to synovial hyperplasia, which involves a variety of immune cells and mediated inflammation. Three RCTs reported MSC treatment of RA. However, due to their different data presentation methods, a systematic review was conducted. Among all RCTs, the use of bone marrow mesenchymal stem cells is generally safe and tolerable. Yang et al. [29] showed that after MSC treatment, the disease activity was weakened and the clinical symptoms (including DAS28) were improved. The improvement of most patients' condition lasts for 12 months, and the total clinical effective rate is 54%. Two patients in the response group had pain and swelling at 24 weeks, and their ESR and CRP levels increased. It is also found that the dosage of prednisone acetate in 23 patients in the experimental group gradually decreased after the intervention. For the immune response, it found that the percentage of CD4+CD 25+Foxp3+Tregs in the response group increased and the percentage of CD4+IL-17A+Th17 cells decreased; and the levels of IL-6 and TNF-*α* decreased significantly.

Shadmanfar et al. [30] shows that MSC may improve the patient's standing time and WOMAC total score and reduce the use of methotrexate and prednisolone. It also showed that patients with knee involvement found that knee pain was reduced by more than 50%. Álvaro-Gracia et al. [31] showed that a moderate proportion of patients meets the comprehensive measure of ACR 20/50/70 response, but fewer patients achieve an improvement of 50% or 70%.

### 3.6. Systemic Lupus Erythematosus

SLE mainly manifests as specific skin lesions, fatigue, weakness, fever, and weight loss and other inflammatory symptoms (such as decreased serum C3). The symptoms of multiple organs are related to the involvement of organs. It was mainly evaluated by SLEDAI. If the kidney is involved, urine protein would be used to assess the kidney involvement.

#### 3.6.1. SLEDAI

**Figure 4:**
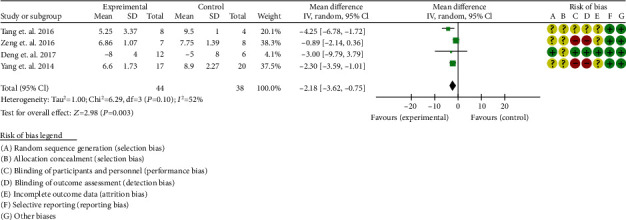
SLEDAI (CI: confidence interval).

Four RCTs reported SLEDAI [32–35]. The heterogeneity test showed that *I*^2^ = 52%, *P* = 0.10, considering the moderate heterogeneity among RCTs. Therefore, the random effects model is used for data analysis. The results show that the SLEDAI in the experimental group was lower than that in the control group (-2.18 (-3.62, -0.75), *P* = 0.003) (Figure 4).

#### 3.6.2. Urine Protein

**Figure 5:**
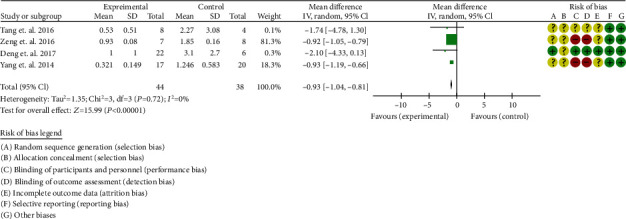
Urine protein (CI: confidence interval).

Four RCTs reported urine protein [32–35]. The heterogeneity test showed that *I*^2^ = 0%, *P* = 0.72, considering the low heterogeneity among RCTs. Therefore, the fixed effects model is used for data analysis. The results show that the urine protein in the experimental group was lower than that in the control group (-0.93 (-1.04, -0.81), *P* < 0.00001) (Figure 5).

#### 3.6.3. Serum C3

**Figure 6:**
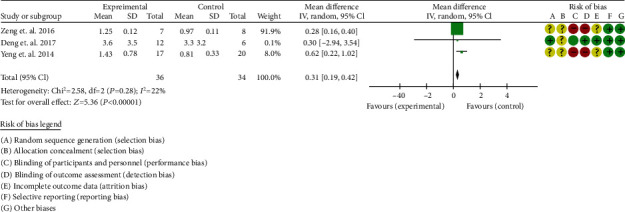
Serum C3 (CI: confidence interval).

Three RCTs reported serum C3 [33–35]. The heterogeneity test showed that *I*^2^ = 22%, *P* = 0.28, considering the low heterogeneity among RCTs. Therefore, the fixed effects model is used for data analysis. The results show that the serum C3 in the experimental group was higher than that in the control group (0.31 (0.19, 0.42), *P* < 0.00001) (Figure 6).

#### 3.6.4. Adverse Events

**Figure 7:**
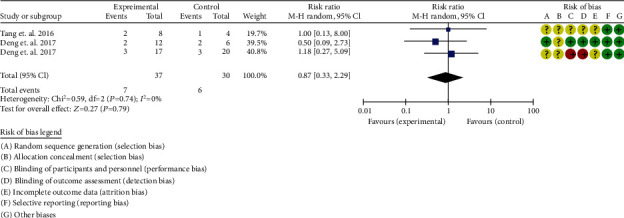
Adverse events of SLE (CI: confidence interval).

Three RCTs reported serum adverse events [32, 34, 35]. The heterogeneity test showed that *I*^2^ = 0%, *P* = 0.74, considering the low heterogeneity among RCTs. Therefore, the fixed effects model is used for data analysis. The results show that the incidence of adverse events between two groups was of no statistical significance (0.87 (0.33, 2.29), *P* = 0.79) (Figure 7).

### 3.7. Inflammatory Bowel Disease

Inflammatory bowel disease is a chronic nonspecific gastrointestinal disease, which is disabling, can seriously affect all aspects of patients' lives, and also causes a heavy burden on the health care system and society. It mainly includes Crohn's disease and ulcerative colitis. Crohn's disease is an inflammatory bowel disease characterized by chronic inflammation of any part of the gastrointestinal tract, with a progressive and destructive course. The clinical symptoms are mainly diarrhea, abdominal pain, blood in the stool, fever, and fatigue. Ulcerative colitis mainly manifests as abdominal pain, rectal pain, bleeding, difficulty in defecation, fever, and fatigue.

#### 3.7.1. Clinical Efficacy

**Figure 8:**
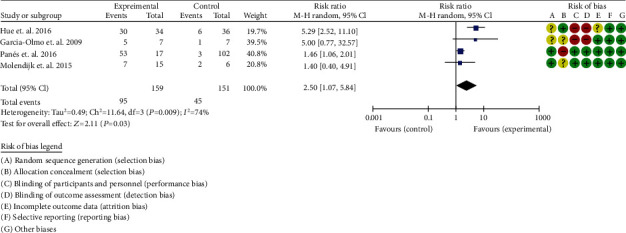
Clinical efficacy (CI: confidence interval).

A total of 4 RCTs were included [36–39]. The heterogeneity test showed that *I*^2^ = 74%, *P* = 0.009, considering the high heterogeneity among RCTs. Therefore, the random effects model is used for data analysis. The results show that the clinical efficacy of the experimental group is better than that of the control group (2.50 (1.07, 5.84), *P* = 0.03) (Figure 8).

#### 3.7.2. Adverse Events

**Figure 9:**
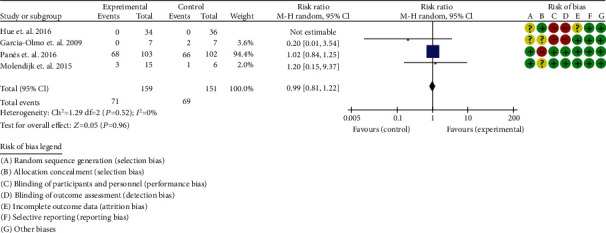
Adverse events of inflammatory bowel disease (CI: confidence interval).

A total of 4 RCTs were included [36–39]. The heterogeneity test showed that *I*^2^ = 0%, *P* = 0.52, considering the low heterogeneity among RCTs. Therefore, the fixed effects model is used for data analysis. The results show that the incidence of adverse events between two groups were of no statistical significance (0.99 (0.81, 1.22), *P* = 0.96) (Figure 9).

### 3.8. Ankylosing Spondylitis

Ankylosing spondylitis mainly manifests as chronic back pain and stiffness. It may be due to erosion, bone growth and vertebral fusion, and inflammatory damage involving Th1/17 and related cytokines. Only one RCT reported the treatment of ankylosing spondylitis with MSC. Su et al. [40] found that compared with the fliximab group (control group), MSC treatment for 6 months may increase the total effective rate; reduce erythrocyte sedimentation rate, intercellular adhesion molecules, and serum TNF-*α*; and improve pain and activity.

### 3.9. Multiple Sclerosis

Multiple sclerosis is an immune disease characterized by chronic demyelination of the central nervous system. In multiple sclerosis patients, monocytes infiltrate into the perivascular space between the arteries and veins and pia mater, axon myelin sheath is lost and destroyed, and glial cell immunoreactivity changes lead to the formation of plaques in multiple parts of the central nervous system. Su et al. [40] found that the progression-free survival (PFS) rate, total number of episodes, and average number of episodes each year in the experimental group were lower than the glucocorticoid group (control group), while the quality of life in the experimental group was higher. Li et al. [44] also showed that compared with the control group, the overall symptoms of MSC-treated patients improved, and the EDSS and recurrence rate were reduced. However, the summary of other outcomes showed different results.

#### 3.9.1. Number of Lesions and Volume of Lesions

**Figure 10:**
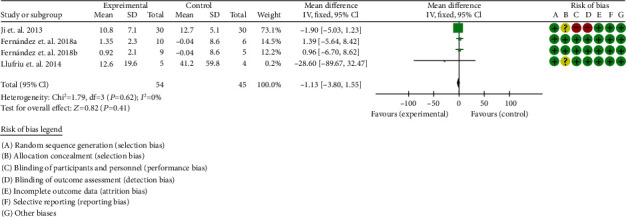
Number of lesions (CI: confidence interval).

Three RCTs reported number and volume of lesions [41, 42, 46]. For a number of lesions, the heterogeneity test showed that *I*^2^ = 0%, *P* = 0.62, considering the low heterogeneity among RCTs. Therefore, the fixed effects model is used for data analysis. The results show that the number of lesions between two groups were of no statistical significance (-1.13 (-3.80, 1.55), *P* = 0.41) (Figure 10).

**Figure 11:**
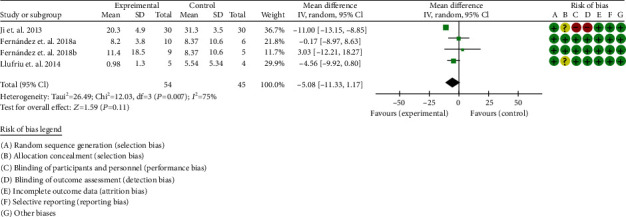
Volume of lesions (CI: confidence interval).

For the volume of lesions, the heterogeneity test showed that *I*^2^ = 75%, *P* = 0.007, considering the high heterogeneity among RCTs. Therefore, the random effects model is used for data analysis. The results show that the volume of lesions between two groups were of no statistical significance (-5.08 (-11.33, 1.17), *P* = 0.11) (Figure 11).

#### 3.9.2. Expanded Disability Status Scale

**Figure 12:**
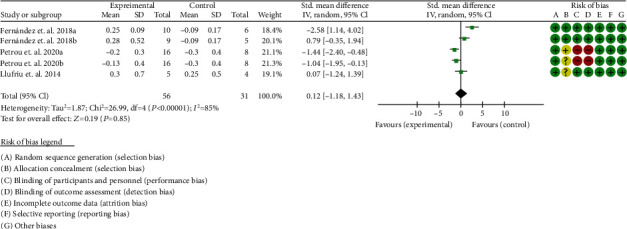
Expanded disability status scale (CI: confidence interval).

Three RCTs reported comparable data of EDSS [42, 43, 46]. The heterogeneity test showed that *I*^2^ = 85%, *P* < 0.0001, considering the high heterogeneity among RCTs. Therefore, the random effects model is used for data analysis. The results show that the EDSS between two groups were of no statistical significance (0.12 (-1.18, 1.43), *P* = 0.85) (Figure 12).

#### 3.9.3. Adverse Events

**Figure 13:**
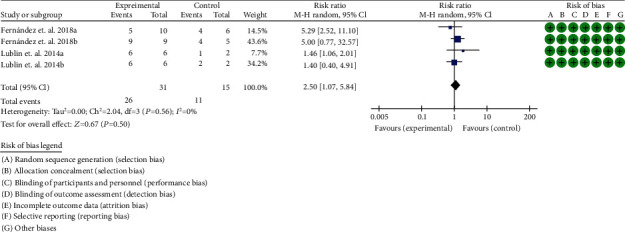
Adverse events of multiple sclerosis (CI: confidence interval).

Two RCTs reported adverse events [42, 45]. The heterogeneity test showed that *I*^2^ = 0%, *P* = 0.56, considering the low heterogeneity among RCTs. Therefore, the fixed effects model is used for data analysis. The results show that the adverse events between two groups were of no statistical significance (1.12 (0.81, 1.53), *P* = 0.50) (Figure 13).

## 4. Discussion

MSCs are a kind of adult stem cells that mainly exist in the bone marrow and have multidifferentiation potential, low immunogenicity, and immunomodulatory properties. In addition to the bone marrow, it can also be isolated and cultured from almost all adult tissues such as the placenta, umbilical cord, cord blood, and adipose tissue. MSCs have powerful immune regulation functions, can induce immune tolerance, and promote hematopoiesis and tissue repair. Studies showed that MSCs have the following characteristics: (1) inhibiting the proliferation of a variety of immune cells including T and B lymphocytes [47], (2) influencing the secretion of cytokines of immune cells to induce their anti-inflammatory effects [48], and (3) it may also release soluble factors and participate in the regulation of rabbit disease [49]. In addition, MSCs do not express major histocompatibility complex (MHC) class I molecules, but mainly express MHC class I molecules, which makes them have low immunogenicity [50]. Due to its multidirectional differentiation potential, immune regulation, hematopoietic support, low immunogenicity, and no immune rejection,, MSCs have been used in the treatment of refractory and severe autoimmune diseases in recent years, providing patients with safe and effective new treatment options.

### 4.1. The Molecular Mechanism of MSC Transplantation in the Treatment of Autoimmune Diseases

MSCs can exert their immunomodulatory properties by inhibiting the proliferation and activation of T lymphocytes, B lymphocytes, natural killer cells (NKs), and dendritic cells (DCs) [51, 52]. Studies have found that the MSCS of patients with autoimmune diseases has many problems such as changes in the number, abnormal cytoskeleton, decreased migration ability, abnormal multidirectional differentiation potential, and abnormal secretion of basic cytokines [53, 54]. It is currently believed that MSCs can inhibit the proliferation of multiple types of allogeneic immune cells [47] and exert immunoregulatory functions on T lymphocytes, B lymphocytes, macrophages, DCs, and NKs [17]. In addition, MSCs may exert immunomodulatory effects by secreting a variety of regulatory cytokines, such as interleukin- (IL-) 4, IL-7, IL-10, *γ*-interferon (IFN-*γ*), and prostaglandin E2 (PGE2) [55].

#### 4.1.1. MSC's Immunomodulatory Effect on T cells

T cells mainly migrate into the thymus from pluripotent stem cells and pre-T cells in the bone marrow, differentiate into mature T cells under the induction of thymus hormone, and then play a series of immune functions. It has the characteristics of participating in delayed-type allergic reactions, regulating transplantation immunity, promoting the formation of precursor cells to produce antibodies, and regulating cellular immunity by secreting a variety of cytokines. It also has different subtypes such as helper, inhibitory, effector, and cytotoxic T cells [56, 57]. Current research has shown that various types of T cells are disordered in patients with autoimmune diseases, and intervention strategies for autoimmune diseases mediated by T cells have become the main direction of new drug development. MSC may secrete a variety of soluble cytokines through paracrine pathways, such as nitric oxide (NO), PGE2, and lumbromine 2,3 dioxygenase (IDO) and nutritional factors such as transforming growth factor (TGF)-*β*3 and tumor necrosis factor (TNF)-*α* [58, 59], to inhibit the proliferation of T lymphocytes [60–63]. This thereby affects the expression of cell surface markers, specific proliferation, the formation of cytotoxic T lymphocytes, Th1 type cell production of INF-*γ*, and Th2 type cell production of IL-4 [64, 65]. Glennie et al. found that MSC may suppress T cells in the G0/G1 phase of the proliferation cycle, downregulate cyclin 22 (an important conversion protein in the G1/S phase), and inhibit the p27Kipl protein, thereby causing a series of changes in the secretion of soluble cytokines, and ultimately inhibiting the activity of T lymphocytes [66]. In addition to cytokines, MSC may also exert an inhibitory effect through direct contact with T lymphocytes. MSC expresses PD-1 ligand 1 (PD-L1) and PD-L2 molecules that bind to programmed death protein 1 (PD-1) on the surface of T lymphocytes, causing the activity of T lymphocytes to be inhibited and hindering their proliferation. These effects may only be exerted when the MSC is in direct contact with T lymphocytes [67]. It can be seen that MSC can affect the immune function of T cells through a variety of mechanisms, and the occurrence and development of a variety of diseases involve abnormal immune regulation of T cells. Therefore, clarifying the immune regulation of MSC to T cells can not only provide a theoretical basis for analyzing its specific mechanism of action in diseases but also provide new ideas for the treatment of immune-related diseases.

#### 4.1.2. MSC's Immunomodulatory Effect on B Cells

When B cells bind to antigens, they activate and proliferate. On the one hand, plasma cells and memory B cells are produced in the germinal center. On the other hand, activating and proliferating B cells cause somatic hypermutation in the variable region of the B cell antigen receptor (BCR), leading to maturation of BCR and antibody affinity, and antibody class switching. This produces plasma cells and memory B cells, which in turn participate in a variety of immune responses [68]. Current research shows that B cells in autoimmune diseases are the main link in the production of autoantibodies, which is the main direction of drug research and development [69]. The negative regulatory effect of MSC on B lymphocytes may be caused by direct contact with B cells to produce a series of cytokines and directly secrete some soluble cytokines to act on B cells. This in turn inhibits the proliferation of B cells and reduces the production of plasma cells and memory B cells, resulting in the reduction of B cells secreting antibodies, cytokines, and chemokines [70]. MSC can also promote the production of granulocyte-macrophage colony-stimulating factor (GM-CSF) through the participation of stem cell antigen 1/lymphocyte antigen 6AIE protein and inhibit the maturation of B lymphocytes. TGF-*β* secreted by MSC participates in the inhibition of B lymphocytes by downregulating or blocking IL-7 derived from stromal cells. MSC can also inhibit B cell secretion of Ig A, lg G, and lg M [71] and downregulate the production of C-x-c motfreceptor 4 (CXCR4) and CXCL13 to inhibit B cell differentiation [70, 72]. Hermankova et al. found that in the presence of IFN-*γ*, MSC may inhibit the proliferation of B lymphocytes by expressing IDO [73].

Therefore, similar to T cells, MSC may regulate the immune function of B cells through a variety of mechanisms and play different roles in a variety of autoimmune diseases.

#### 4.1.3. MSC's Immunomodulatory Effect on Immune Dendritic Cells (DC)

DC can efficiently ingest, process, and present antigens and is the body's strongest antigen-presenting cell. Mature DC can activate initial T cells and then initiate, regulate, and maintain immune response, while immature DC has strong migration ability, can quickly migrate to the lesion site, and participates in the immune response [74]. The change of DC may damage the immune regulation mechanism, break the balance of natural immune tolerance, and cause autoimmune diseases. In addition, the activation of T cells and B cells by DC is also closely related to the occurrence of autoimmune diseases. Therefore, it is believed that DC is the hub of the pathological pathway of autoimmune diseases. The related research of DC on the treatment of autoimmune diseases illustrates the close relationship between DC and autoimmune diseases from another angle [75, 76].

When MSC and DC are cocultured, it can inhibit the differentiation of monocytes into DC by downregulating the expression of CD1a, CD86, and HLA-DR of MHC class II molecules. It also inhibits the expression of CD83, inhibits the secretion of TNF-4 from DC1 cells, and enhances the secretion of IL-10 from DC2 [77], thereby changing the DC phenotype from mature to immature stage, leading to immune silence [78]. MSC can significantly inhibit the transformation of GM-CSF and L-4 leads from CD14+ monocytes to DC. Djouad et al. found that MSC can secrete IL-6 and downregulate the expression of MHC I molecules, CD40, and CD86 on the surface of mature DC, or by secreting TGF-*β*, PGE2 and other cytokines, inhibit the activity of DC, and cause DC to differentiate into immature phenotype [79]. MSC affects the maturation of DC through a variety of ways, including the expression of antigen and costimulatory molecules, changes in antigen presentation and migration ability, maintaining the expression of cadherin, and inhibiting the expression of Cc motfireceptor 7 (CCR7) and Cc motiligand 19 (CCL19), thereby inhibiting the migration of DC and so on [72]. Therefore, MSC may inhibit the generation, proliferation, antigen presentation, migration, and deformation ability of DC and participate in the differentiation and maturation of DC.

In summary, it is currently believed that MSCs exert their immune regulation function mainly by inhibiting the proliferation of T lymphocytes, inhibiting the proliferation and differentiation of B lymphocytes, regulating the activity of NKs, and preventing the maturation of DCs. In the future, more MSCs' immune regulation mechanisms would be revealed.

### 4.2. Clinical Evidence of MSC Transplantation in the Treatment of Autoimmune Diseases

#### 4.2.1. RA

Animal studies have shown that intraperitoneal injection of MSCs can effectively alleviate the symptoms of arthritis in mice [80]. A variety of MSC transplantation treatments, such as bone marrow source, fat source, and cord blood source, can effectively alleviate the symptoms of RA model mice [81, 82]. Previous studies have suggested that due to lack of immunogenicity and significant local immunosuppressive ability, MSCs from umbilical cord matrix tissue can be used more safely in allogeneic transplantation and can exert their immunomodulatory effects in the body without prior induction and activation and has gradually replaced bone marrow-derived MSCs [83]. The specificity of umbilical cord MSCs may be due to differences in gene and protein expression profiles, that is, increased expression of immunomodulatory surface proteins, such as CD200, CD273, and CD274, and cytokines such as IL-1*β*, IL-8, leukemia inhibitory factor, and TGF-*β*2 [84]. MSCs inhibit the proliferation of T lymphocytes and reduce the expression levels of INF-*γ* and TNF-*α*, thereby improving the clinical symptoms of autoimmune encephalomyelitis model mice. In addition, MSCs can accumulate in peripheral immune organs, causing immune tolerance to peripheral T lymphocytes [85, 86]. Studies have found that TGF-*β* and IL-4 are also involved in the immune regulation of MS by MSCs [87, 88].

The 3 RCTs included in this systematic review showed the therapeutic effect of MSC transplantation on RA. Yang et al. (2018) showed that after MSC treatment, the disease activity was weakened and the clinical symptoms (including DAS28) were improved. It also found that the dosage of prednisone acetate in 23 patients in the experimental group gradually decreased after the intervention. For the immune response, it found that the percentage of CD4+CD 25+Foxp3+Tregs in the response group increased and the percentage of CD4+IL-17A+Th17 cells decreased; and the levels of IL-6 and TNF-*α* decreased significantly. Shadmanfar et al. (2018) shows that MSC may improve the patient's standing time and WOMAC total score and reduce the use of methotrexate and prednisolone. Álvaro-Gracia et al. (2017) showed that a moderate proportion of patients meets the comprehensive measure of ACR 20/50/70 response, but fewer patients achieve an improvement of 50% or 70%. In addition, the combination therapy of mesenchymal stem cells and other cytokines will become a new mesenchymal stem cell combination strategy in the future. He et al. through intravenous injection of IFN-*γ* to patients, “emerging” mesenchymal stem cells, forming an immune microenvironment that is conducive to mesenchymal stem cells to exert their anti-inflammatory and immune regulation functions, to treat autoimmune inflammatory diseases such as RA [89]. Compared with the treatment of mesenchymal stem cell transplantation alone, during the three-month clinical observation period, the effective rate of “empowering” mesenchymal stem cell transplantation in the treatment of rheumatoid arthritis has been significantly improved, from 53.3% to 93.3%. This research had become an important advancement in the field of mesenchymal stem cell treatment of rheumatoid arthritis in recent years [89]. At present, the team is conducting a multicenter clinical randomized trial to prove the effect of the therapy in the treatment of diseases such as RA and SLE.

For dosage and infusion method, Yang et al. (2018) and Álvaro-Gracia et al. (2017) use intravenous infusion, and the dose of MSC is different. Shadmanfar et al. (2018) used the intra-articular injection method but did not describe the specific dosage. All three have curative effects, but because the same indicators are not reported, they cannot be combined for meta-analysis. And because the RCTs with the same dose and infusion methods were few, subgroup analysis was hard to perform. Therefore, it is not yet known which dose and which intervention method works best. We may only speculate based on current evidence that 1 to 3^∗^10^7^ cells (or 1^∗^10^6^ cells/kg dose) may achieve therapeutic effects through intravenous infusion or intra-articular injection.

#### 4.2.2. SLE

SLE is an autoimmune disease that mainly manifests itself in the formation of autoantibodies and involves multiple organs and multiple systems. SLE is common in women of childbearing age, and its clinical manifestations are complex and diverse, and the exact pathogenesis has not been confirmed. At present, the main treatment options for SLE are glucocorticoids and immunosuppressive agents. This program has poor curative effect on some patients with refractory lupus and has many adverse reactions, which has a greater impact on the quality of life of patients. Animal studies have shown that the MRL/lpr effect of MSC alone or combined with cyclophosphamide in the treatment of SLE model mice is better than cyclophosphamide alone, which is shown in reversing multiple organ dysfunction in lupus mice and improving proteinuria and renal pathological damage [90, 91]. In addition, studies have confirmed that MSCs from different sources can control disease progression and improve disease performance in lupus model mice. Cord blood-derived MSCs can also effectively relieve the condition of lupus model mice [92], and fat-derived MSCs can improve the immune system damage caused by lupus to a certain extent and can reduce kidney damage [93].

This meta-analysis showed that the SLEDAI and urine protein in the experimental group was lower than that in the control group. The serum C3 in the MSC group was also higher than that in control group. In terms of safety, there was no statistical difference in the incidence of adverse events between the MSC group and the control group. It can be considered that the safety of MSC and the control group (placebo or traditional therapy) is equivalent. Other clinical trials also showed that MSC transplantation has significant clinical therapeutic effects, which can stabilize the patient's condition and reduce the recurrence of the patient's condition. The patients received MSC transplantation without rejection, and MSCs have good safety [90, 94]. Through a multicenter clinical study on MSC transplantation for the treatment of SLE, a total of 40 patients from 4 centers were enrolled. The results of the study showed that the overall survival rate after transplantation was 92.5%, and no serious transplant-related adverse reactions occurred [95]. Long-term follow-up of 9 patients with refractory SLE for up to 6 years showed that there was no increase in serum tumor markers before and 6 years after MSC infusion [95]. It shows that in these patients, allogeneic umbilical cord-derived MSC transplantation has good safety. In summary, combined with single-arm clinical trials and RCTs, for refractory SLE, MSC transplantation treatment has good safety.

For dosage and infusion method, except for the renal artery method used by Zeng et al. (2016), the intravenous infusion method is used for other RCTs. And these RCTs use different doses (from 1^∗^10^6^ cells to 2^∗^10^8^ cells). Therefore, it is difficult to evaluate which dose and method of administration are better. We may only speculate based on current evidence that 1^∗^10^6^ cells to 2^∗^10^8^ cells MSC transplantation may achieve therapeutic effects through intravenous infusion or renal artery.

#### 4.2.3. Inflammatory Bowel Disease

Immune dysfunction is believed to play a key role in the occurrence and development of ulcerative colitis. Research suggests that mesenchymal stem cells may help tissue regeneration by suppressing inappropriate immune responses and providing various cytokines instead of directly restoring damaged cells [96]. The pathogenesis of ulcerative colitis is unclear. Studies have found that in the intestinal mucosa of patients with active ulcerative colitis, there is a cytokine storm, especially IL 17 levels are significantly increased [97]. The imbalance in the ratio of regulatory T cells (Tregs)/helper T cells 17 (Th17) may be related to the occurrence and development of ulcerative colitis. Only CD4+CD25+regulatory T cells expressed by Foxp3 have immunomodulatory effects. The combination of Foxp3 and nuclear receptors can significantly inhibit the transcription of interleukin 17, thereby affecting the differentiation of Th17 cells [98]. Studies have found that Rab27A and Rab27B are GTPases related to exosomes, which are related to the secretion of exosomes and their docking in the plasma membrane of various cells. Compared with the healthy control group, a significant increase in the number of Rab27A+ or Rab27B+ intestinal immune cells can be observed in the colonic mucosa of the active ulcerative colitis group. This indicates that the immune response mediated by exosomes plays an important role in the pathogenesis of ulcerative colitis [99]. MSC can induce the apoptosis of T lymphocytes by secreting exosomes, stimulate monocytes to secrete IL10 and TGF *β*, promote the upregulation of CD4+ CD25+ Foxp3+ regulatory T cells, reduce the level of inflammatory factor IL 4, and increase the level of anti-inflammatory factor IL 10 to regulate the immune response. Anti-inflammatory factors such as TGF *β* and IL 10 can stimulate mesenchymal stem cells in vitro to secrete exosomes more effectively, which in turn promotes the upregulation of regulatory T cells, reduces intestinal inflammation, and promotes the repair and regeneration of damaged tissues [100, 101]. In addition, animal experiments have shown that mesenchymal stem cells can migrate to the colon and differentiate into vascular endothelial cells to promote the formation of new blood vessels in damaged parts [102–104], promote the reconstruction of microcirculation, and thus facilitate the repair of colonic mucosal inflammation. The number of directional migration of stem cells is related to the degree of tissue damage. With the aggravation of the damage, the migration rate of mesenchymal stem cells increases, and the number in the recovery period decreases significantly [105, 106]. When inflammation occurs in the intestine, mesenchymal stem cells can migrate in the body and settle on the surface of the intestinal mucosa and proliferate and differentiate into new colonic mucosal epithelial cells to repair the injured site [107]. Brittan et al. found that MSC transplantation can colonize and differentiate into intestinal subepithelial myofibroblasts after transplantation and promote intestinal mucosal repair and neovascularization by improving the intestinal microenvironment [108]. In the human body, whether mesenchymal stem cells differentiate directly into intestinal mucosal epithelial cells or myofibroblasts and promote intestinal epithelial cell repair and angiogenesis by improving the intestinal microenvironment still needs further research to confirm. This meta-analysis found that it can improve clinical efficacy. The incidence of adverse events between two groups was of no statistical significance.

For the dosage and infusion method, Garcia-Olmo et al. (2009), Panés et al. (2016), and Molendijk et al. (2015) used local injection methods, while Hu et al. (2016) used intravenous infusion. As intravenous infusion administration methods are reported less, it is not known which route of administration is better. And since the doses administered are also diverse, it is not known which method of administration is better. We may only speculate based on current evidence that 1 to 5^∗^10^7^ cells for MSC transplantation may achieve therapeutic effects through intravenous infusion or local injection.

#### 4.2.4. Multiple Sclerosis

Ji et al. (2013) found that the progression-free survival (PFS) rate, total number of episodes, and average number of episodes each year in the experimental group were lower than that in the glucocorticoid group (control group), while the quality of life in the experimental group was higher. However, the summary of other outcomes showed that the number and volume of lesions and EDSS between the experimental group and control group was of no statistical significance. This controversial result is interesting, so more relevant research is needed in the future to amend or confirm the conclusion. However, basic research has found that MSC may have the effect of treating multiple sclerosis.

Multiple sclerosis is a chronic inflammatory demyelinating disease that mainly affects the central nervous system. Its pathological characteristics are mainly manifested by cell infiltration of myelin-specific autoreactive T cells and subsequent neuroinflammatory response, demyelination response, and neuronal cell damage. The destruction of axon integrity and the accumulation of irreversible sclerosis are the main causes of irreversible neurological damage [109, 110]. The pathogenesis of multiple sclerosis involves a variety of cells in innate immunity, such as Th17 helper T cells 1, Treg, microglia, dendritic cells, and macrophages. The destruction of the balance between helper T cells 1 and helper T cells 17 is considered to be an important mechanism leading to the pathogenesis of multiple sclerosis, and regulatory T cells are considered to be a key regulator of the adaptive immune response of multiple sclerosis [111, 112].

Although there are many kinds of drugs that can be used for the treatment of multiple sclerosis, most of them can only control the progression of the disease and improve the clinical symptoms of patients, but they cannot completely cure the disease. Once the patient's clinical manifestations develop into progressive disability, there is no effective way to protect, repair, and regenerate nerve tissue to restore the patient's nerve function. Therefore, myelin and nerve cell regeneration are still the main obstacles to the treatment of multiple sclerosis [113, 114]. In the past 20 years, stem cell transplantation has been considered a potentially effective treatment for invasive multiple sclerosis [115], and different types of stem cells, even stem cells of the same type but from different sources, have their unique characteristics.

Mesenchymal stem cells exert their therapeutic effects on multiple sclerosis mainly by regulating the immune response and promoting nerve repair. The regulation effect of rabbit disease is manifested by inhibiting innate and adaptive immune response, inhibiting the proliferation of pathogenic effect CD4+ T cells and B cells, regulating CD8+ T cell subsets, inducing the generation of regulatory T cells, and affecting the functions of dendritic cells and natural killer cells. The nerve repair function is produced by secreting a variety of neurotrophic factors, affecting the differentiation of neural stem cells, and promoting remyelination and axon survival [115, 116]. Barati et al. found that promoting the production of M2 type microglia and inhibiting the expression of proinflammatory cytokines may be the mechanism for mesenchymal stem cells to treat demyelinating diseases [117]. Bone marrow mesenchymal stem cells can improve the symptoms of patients with multiple sclerosis by inhibiting the inflammatory response in the central nervous system, regulating the expression of interleukin 6, stimulating the production of nerve growth factor, and protecting axons [118]. Wang et al. showed that the supernatant of bone marrow mesenchymal stem cells can affect the function of CD4+ T cells [64]. It thereby inhibits the secretion of inflammatory factors in the peripheral blood of experimental autoimmune encephalomyelitis and reduces the degree of demyelination in the central nervous system of mice with experimental autoimmune encephalomyelitis.

Compared with human bone marrow mesenchymal stem cells, mesenchymal stem cells derived from human embryonic stem cells can significantly reduce the clinical symptoms of experimental autoimmune encephalomyelitis and more effectively prevent demyelination. This difference may be related to the high permeability of mesenchymal stem cells derived from human embryonic stem cells [64]. In addition to bone marrow mesenchymal stem cells, adipose-derived mesenchymal stem cells are also commonly used to treat multiple sclerosis and experimental autoimmune encephalomyelitis. Adipose-derived mesenchymal stem cells can pass through the blood-brain barrier and reduce the infiltration of brain B cells, T cells, and macrophages. In experimental autoimmune encephalomyelitis mice treated with adipose-derived mesenchymal stem cells, human leukocyte antigen G is one of the main factors to reduce the severity of the disease [119]. In addition, Li et al. [120] found that adipose-derived mesenchymal stem cells can also reduce the Th17/Treg ratio by releasing leukemia inhibitory factors and reduce the degree of disability in experimental autoimmune encephalomyelitis. Kurte et al. [121] observed that transplantation of mesenchymal stem cells before the onset of the disease in experimental autoimmune encephalomyelitis mice or at the peak of the disease has the best therapeutic effect. The findings of Strong et al. [122] emphasize the importance of choosing a donor. They injected adipose-derived mesenchymal stem cells from obese and wasting donors into mice with experimental autoimmune encephalomyelitis by intraperitoneal injection. The results showed that adipose-derived mesenchymal stem cells from obese donors failed to inhibit inflammation and clinical symptoms, and adipose-derived mesenchymal stem cells from obese donors increased the secretion of proinflammatory cytokines. Cell transplantation through intravenous injection is usually the preferred injection method in experiments, but intranasal administration can bypass the blood-brain barrier and directly enter the brain through the olfactory and trigeminal nerve pathways, which also provides researchers with another option [123].

For the dosage and infusion method, RCTs use different doses (from 5^∗^10^7^ cells to 6^∗^10^8^ cells). Therefore, it is difficult to evaluate which dose and method of administration are better. We may only speculate based on current evidence that 5^∗^10^7^ cells to 6^∗^10^8^ cells MSC transplantation may achieve therapeutic effects through intrathecal injection or intravenous infusion.

#### 4.2.5. Ankylosing Spondylitis

Ankylosing spondylitis is an autoimmune disease mediated by immune complexes. The main symptom is a chronic progressive inflammatory disease that invades the spine and affects the patient's sacroiliac joints and surrounding joint tissues. It has a high clinical morbidity and disability rate. The study found that, compared with healthy donors, although bone MSCs (BMSCs) obtained from patients showed normal proliferation, cell viability, surface markers, and multiple differentiation characteristics, and their immunomodulatory ability was significantly reduced [124]. Xie et al. [125] found that because BMSCs secreted more bone morphogenetic protein 2 (BMP2) and less noggin (NOG), BMSCs of AS patients had stronger osteogenic differentiation ability than BMSCs of normal donors. This state may contribute to the underlying pathological osteogenesis in AS. Animal studies have shown that after MSCs are injected into mice, Th17 cells are inhibited and the percentage of CD4+-CD25+-Foxp3+-Treg cells increases [126]. In addition, AS patients have a low number of Treg cells, a low B cell level, and abnormal function [127]. Studies have shown that MSCs can differentiate T cells into Th2 phenotype and inhibit the differentiation of Th17 cells, thereby reducing the cytokine levels of Th17 cells and promoting the regeneration process of subsequent tissue damage [128]. Clinical trials have shown that MSCs may help relieve the symptoms of AS patients [127–129]. Wang et al. [130] found that the Bath ankylosing spondylitis disease activity index (BASDAI), night pain score (VAS) and Bath ankylosing spondylitis functional index (BASFI) improved. Wang et al. [130] found that the Bath ankylosing spondylitis disease activity index (BASDAI), night pain score (VAS), and Bath ankylosing spondylitis functional index (BASFI) improved. Patients' ESR and immunoglobulin G decreased significantly at 3, 6, and 12 months after stem cell transplantation. This systematic review only found one RCT related to the treatment of ankylosing spondylitis with MSC. It is found that compared with the fliximab group, MSC treatment for 6 months may increase the total effective rate; reduce erythrocyte sedimentation rate, intercellular adhesion molecules, and serum TNF-*α*; and improve pain and activity.

For the dosage and infusion method, one RCT is injected with 1^∗^10^6^ cells/kg through intravenous infusion, and it has a certain effect. For multiple sclerosis, the administration methods and dosages of each RCTs are varied, and the summary results have no significant curative effect compared with the control group. Therefore, the optimal dosage and route of administration are not yet known. We may only speculate based on current evidence that 1^∗^10^6^ cells/kg dose of MSC transplanted by the intravenous infusion method has not been able to observe the therapeutic effect.

Nevertheless, more RCTs are still needed to further determine the key points of MSCs in the treatment of ankylosing spondylitis, such as cell source, dosage, route of drug administration, and especially intervention in the most ideal disease stage (early or late).

## 5. Conclusion

This systematic review and meta-analysis summarized the safety and effectiveness of MSC in the treatment of autoimmune diseases (RA, SLE, inflammatory bowel disease, multiple sclerosis, and ankylosing spondylitis) and provides relevant evidence for the future clinical research design (such as dose and disease severity) of clinical trials for MSC treatment of autoimmune diseases (such as rheumatoid arthritis, SLE, inflammatory bowel disease, multiple sclerosis, and ankylosing spondylitis).

## Figures and Tables

**Table 1:** The characteristics of the included studies.

Disease	Study	Trial registration number	Country	Sample size (female/male)		Intervention		Dosage	Route of medication	Relevant outcomes	Mean age (years)		Disease duration (years)		Duration
				Trial group	Control group	Trial group	Control group				Trial group	Control group	Trial group	Control group	
Rheumatoid arthritis	Yang et al. 2018 [29]	ChiCTR-ONC-16008770	China	52 (40/12)	53 (43/10)	MSC injection	1% albumin in physiological saline as the treatment or 50 mL of 1% albumin in normal saline without UCMSCs	1^∗^10^6^ cells/kg	Intravenous infusion	Disease activity, adverse events	Response: 50.7; nonresponse: 51.2	49.8	Response: 4 ± 2.75; nonresponse: 3.94 ± 2.79	3.89 ± 2.52	48 weeks
	Shadmanfar et al. 2018 [30]	NCT01873625	Iran	13 (13/0)	15 (13/2)	MSC injection	Normal saline	Not known	Intra-articular injection	Disease activity, adverse events	50.4 ± 8.5	48.1 ± 10.8	—	—	48 weeks
	Álvaro-Gracia et al. 2017 [31]	NCT01663116	Spain	46 (42/4)	7 (6/1)	MSC injection	Ringer's lactate solution	1^∗^10^7^ cell or 2∗10^7 cell or 3∗10^7 cell	Intravenous infusion	Disease activity, adverse events	54.15 ± 7.79	58.3 ± 14.25	14.36 ± 6.60	22.73 ± 22.65	24 weeks
Systemic lupus erythematosus	Tang et al. 2016 [32]	—	China	12 (11/1)		MSC injection+prednisolone+cyclophosphamide+mycophenolate	Prednisolone+cyclophosphamide+mycophenolate	5^∗^10^7^ cells	Intravenous infusion	Systemic lupus erythematosus disease activity index (SLEDAI), urine protein, adverse events	19-44		0.2-14		24 weeks
	Zeng et al. 2016 [33]	—	China	7 (not known)	8 (not known)	MSC injection+glucocorticoid+mycophenolate mofetil	Glucocorticoid+mycophenolate mofetil	1^∗^10^6^ cells	Renal artery	SLEDAI, urine protein, C3	—	—	—	—	48 weeks
	Deng et al. 2017 [34]	NCT01539902	China	12 (11/1)	6 (6/0)	MSC injection	Placebo	2^∗^10^8^ cells	Intravenous infusion	SLEDAI, urine protein, C3, adverse events	29 ± 10	29 ± 7	4.92 ± 3.67	7.83 ± 4.58	48 weeks
	Yang et al. 2014 [35]	—	China	17 (15/2)	20 (20/0)	MSC injection+glucocorticoid+cyclophosphamide	Glucocorticoid+cyclophosphamide	3^∗^10^7^ cells	Intravenous infusion	SLEDAI, urine protein, C3, adverse events	35.22 ± 10.13	36.23 ± 10.67	4.01 ± 2.97	4.31 ± 3.77	48 weeks
Inflammatory bowel disease	Hu et al. 2016 [36]	NCT01221428	China	34 (13/21)	36 (14/22)	Mesenchymal stem cell (MSC) infusions twice besides the base treatment with a 7 day interval	Normal saline infusions twice besides the base treatment with a 7-day interval	3.8 ± 1.6^∗^10^7^ cell	Intravenous infusion	Clinical efficacy (based on Mayo scores), Mayo score and IBDQ score, adverse events	42.9 ± 23.1	43.7 ± 28.7	5.6 ± 4.2	6.1 ± 4.9	24 weeks
	Garcia-Olmo et al. 2009 [37]	—	Spain	14 (11/3)		Adipose-derived stem cells+fibrin glue	Fibrin glue only	1^∗^10^7^ cell/mL	Local injection	Clinical efficacy (healing of a complex perianal fistula), quality of life score (SF-12), adverse events	43.99 ± 8.97		—	—	8 weeks
	Panés et al. 2016 [38]	NCT01541579	Seven European countries and Israel	107(47/60)	105(49/56)	MSC injection	Normal saline injection	1.2^∗^10^7^ cell	Local injection	Clinical efficacy, adverse events	39.0 ± 13.1	37.6 ± 13.1	12.1 ± 10.0	11.3 ± 8.9	24 weeks
	Molendijk et al. 2015 [39]	NCT01144962	Netherlands	15 (6/9)	6 (3/3)	MSC injection	Normal saline+human albumin injection	1^∗^10^7^ cell or 3^∗^10^7^ cell or 9^∗^10^7^ cell	Local injection	Clinical efficacy (healing of a perianal fistula), adverse events	21-54	27-49	5-28	1-20	12 weeks
Ankylosing spondylitis	Su et al. 2020 [40]	—	China	20(8/12)	20(7/13)	MSC injection	Fliximab injection	1^∗^10^6^ cells/kg	Intravenous infusion	Clinical efficacy, immune index, and adverse events	32.15 ± 2.33	32.12 ± 2.31	—	—	24 weeks
Multiple sclerosis	Ji et al. 2013 [41]	—	China	60 (39/19)		MSC injection+rituximab	Glucocorticoid	5^∗^10^7^ cells	Intravenous infusion and oral	Progression-free survival (PFS) rate, number of episodes, ability of daily living (ADL) scale, number of lesion, volume of lesion (cm^3^), adverse events	28.3 ± 4.5		2.93 ± 0.13		96 weeks
	Fernández et al. 2018 [42]	NCT01056471	Spain	19 (13/6)	11 (8/3)	MSC injection low and high dose	Ringer's lactate	1^∗^10^6^ cells/kg or 4^∗^10^6 cells/kg	Intravenous infusion	Expanded disability status scale (EDSS), number of lesion, volume of lesion (cm^3), adverse events	Low dose: 44.8 ± 8.0; high dose: 47.8 ± 9.7	46.3 ± 8.9	Low dose: 15.4 ± 6.1; high dose: 18.7 ± 8.7	18.9 ± 7.3	24 weeks
	Petrou et al. 2020 [43]	NCT02166021	Israel	32 (16/16)	16(4/12)	MSC injection	Normal saline	1∗10^6^ cells/kg	Intrathecal injection or intravenous infusion	EDSS, adverse events	Intravenous infusion: 49.05 ± 7.2; intrathecal injection: 47.42 ± 10.4	45.89 ± 10.9	Intravenous infusion: 10.28 ± 4.48; intrathecal injection: 12.90 ± 8.74	14.94 ± 8.27	24 weeks
	Li et al. 2014 [44]	—	China	13 (9/4)	10 (7/3)	MSC injection	No normal saline	4^∗^10^6^ cells/kg	Intravenous infusion	EDSS, cytokine	41.7 ± 5.6		2.90 ± 0.9		6 weeks
	Lublin et al. 2014 [45]	—	The United States and Canada	12(9/3)	4 (2/2)	MSC injection	Placebo	1.5^∗^10^8^ cells or 6^∗^10^8^ cells	Intravenous infusion	EDSS, adverse events	36-58	40-52	—	—	48 weeks
	Llufriu et al. 2014 [46]	NCT01228266	Spain	9(7/2)		MSC injection	Suspension media	1 − 2^∗^10^6^ cells/kg	Intravenous infusion	EDSS, number of lesion, volume of lesion (cm^3^), adverse events	36.8 ± 8.4		8.1 ± 2.15		24 weeks

## Data Availability

All data generated or analyzed during this study are included in this published article.

## Supplementary Materials

Supplementary Materials PRISMA 2020 checklist: checklist. Table S1: search strategies for PubMed and Embase.
